# MADS-Box Genes and Gibberellins Regulate Bolting in Lettuce (*Lactuca sativa* L.)

**DOI:** 10.3389/fpls.2016.01889

**Published:** 2016-12-16

**Authors:** Yingyan Han, Zijing Chen, Shanshan Lv, Kang Ning, Xueliang Ji, Xueying Liu, Qian Wang, Renyi Liu, Shuangxi Fan, Xiaolan Zhang

**Affiliations:** ^1^Plant Science and Technology College, Beijing University of Agriculture/New Technological Laboratory in Agriculture Application in BeijingBeijing, China; ^2^Beijing Key Laboratory of Growth and Developmental Regulation for Protected Vegetable Crops, Department of Vegetable Sciences, China Agricultural UniversityBeijing, China; ^3^Shanghai Center for Plant Stress Biology, Shanghai Institutes for Biological Sciences, Chinese Academy of SciencesShanghai, China

**Keywords:** lettuce, bolting, flowering integrators, RNA-Seq, proteomics

## Abstract

Bolting in lettuce is promoted by high temperature and bolting resistance is of great economic importance for lettuce production. But how bolting is regulated at the molecular level remains elusive. Here, a bolting resistant line S24 and a bolting sensitive line S39 were selected for morphological, physiological, transcriptomic and proteomic comparisons. A total of 12204 genes were differentially expressed in S39 vs. S24. Line S39 was featured with larger leaves, higher levels of chlorophyll, soluble sugar, anthocyanin and auxin, consistent with its up-regulation of genes implicated in photosynthesis, oxidation-reduction and auxin actions. Proteomic analysis identified 30 differentially accumulated proteins in lines S39 and S24 upon heat treatment, and 19 out of the 30 genes showed differential expression in the RNA-Seq data. Exogenous gibberellins (GA) treatment promoted bolting in both S39 and S24, while 12 flowering promoting MADS-box genes were specifically induced in line S39, suggesting that although GA regulates bolting in lettuce, it may be the MADS-box genes, not GA, that plays a major role in differing the bolting resistance between these two lettuce lines.

## Introduction

The full life cycle of higher plants includes two stages: vegetative growth and reproductive growth. Bolting and flowering is the key transition from the vegetative to reproductive phase, and is regulated by many genetic pathways responding to endogenous cues and environmental factors, including photoperiod, vernalization, gibberellin (GA), autonomous and age pathways (Mouradov et al., 2002; Komeda, 2004). In the *Arabidopsis thaliana*, ∼180 genes have been implicated in flowering-time control according to isolation of loss-of-function mutants or analysis of transgenic plants (Komeda, 2004; Fornara et al., 2010). For examples, *FLOWERING LOCUS T (FT), SUPPRESSOR OF OVEREXPRESSION OF CO 1 (SOC1)* and *LEAFY (LFY)* act as the major flowering integrators that determine the eventual flowering time in *Arabidopsis* (Mouradov et al., 2002; Parcy, 2005). The *FT* mRNA is expressed in mature leaf, and its protein is transported to shoot apical meristem (SAM) to interact with FLOWERING LOCUS D (FD), and the resultant FT-FD complex induces the expression of several downstream genes such as *APETALA1(AP1)*, *LFY*, and *FRUITFULL (FUL)* (Ruiz-Garcia et al., 1997; Benlloch et al., 2011). *SOC1* encodes a MADS-box protein that integrates multiple flowering signals derived from photoperiod, temperature, hormone and age-related pathways (Lee and Lee, 2010). SOC1 interacts with multiple MADS-box proteins, including FUL, AP1 and AGAMOUS LIKE24 (AGL24), and regulates several flowering genes, such as *SHORT VEGETATIVE PHASE (SVP), AGL15* and *AGL18*, by directly binding to their regulatory sequences (Komeda, 2004; Liu et al., 2008; Melzer et al., 2008; Torti and Fornara, 2012; Balanza et al., 2014). *LFY* is a floral meristem identity gene that promotes the floral transition as well (Araki, 2001). During the vegetative phase, *LFY* is expressed in the leaf primordia and is regulated by both photoperiod and Gibberellin (Simon et al., 1996; Blazquez and Weigel, 2000).

Gibberellins (GAs) are a category of plant hormones that regulate various ways of plant growth and development such as seed germination, leaf expansion, stem elongation and flower development through promoting cell division and cell elongation (Blazquez et al., 1998; Richards et al., 2001). Despite that GAs have been shown to regulate the transition to flowering, the specific roles of GAs in flowering vary in different circumstances and different species. For example, the abundance of endogenous GAs positively correlates with conditions that promote flowering, and exogenous GA application can induce flowering in many plants such as spinach, apple tree, *Lolium temulentum* and *Chrysanthemum* (Looney et al., 1985; King et al., 2003; Yang et al., 2014). However, applied GAs usually inhibit flowering of woody angiosperms, and has no effect on flowering in *Salvia hispanica* L. (Bernier et al., 1993; Levy and Dean, 1998). Gibberellins have been shown to promote flowering of *Arabidopsis* by activating the LEAFY promoter, and crosstalk with photoperiod and vernalization pathways (Zanewich and Rood, 1995; Blazquez et al., 1998; Yu et al., 2004).

Flowering at the proper time of the year is a key factor for successful reproduction and is of great commercial significance for crops and ornamental plants (Xiao et al., 2012; Yang et al., 2014). Premature bolting and flowering is an undesirable agricultural trait that causes great economic loss in vegetables such as lettuce, cabbage and radish (Yoshida et al., 2010; Xiao et al., 2012; Nie et al., 2016). Lettuce (*Lactuca sativa* L.) belongs to the Asteraceae family and is the most popular leafy vegetable that is cultivated worldwide and consumed during its vegetative growth (Fukuda et al., 2009). In 2013, the cultivating area of lettuce and endive was 1148 kha in the world with the production of 24896 ton^1^. Lettuce is a diploid, self-pollination species with 2n = 2x = 18 chromosomes. Based on plant morphology, lettuce can be classified into four types including iceberg lettuce, romaine lettuce, butterhead lettuce and non-heading leaf-type lettuces (Simko et al., 2013). Unlike most other flowering plants, transition from vegetative to reproductive phase in lettuce is induced by high temperatures, and followed by rapid stem elongation (bolting) and flowering (Fukuda et al., 2009). Once bolted, leafy lettuce loses its marketability and thus bolting is a serious problem for production all year around, especially during the hot summer.

With recent advances in sequencing technologies, genomic and transcriptomic data are dramatically increasing, and it is now conceivable to combine genomic and transcriptomic data with proteomic results for large-scale gene expression and protein characterization (Li et al., 2016). For examples, in cucumber, time course transcriptome analyses of corolla indicated that cytokinin and nutrition played essential roles for the delayed flower opening in super ovary (Sun et al., 2016). Transcriptional sequencing has also been used to clarify the gene expression patterns during floral development in bamboo (*Dendrocalamus latiflorus*) (Zhang et al., 2012), to identify the genes associated with flowering time in *Oncidium* (Chang et al., 2011), and to explore the floral odor related genes in *Phalaenopsis* (Hsiao et al., 2006). In soybean (*Glycine max*), proteomic comparison of flower bud and flower has revealed that 29 proteins related to mitochondrial protein transport and assembly, secondary metabolism, and pollen tube growth were differentially upregulated during flower development (Ahsan and Komatsu, 2009).

In this study, two leafy lettuce lines with different bolting resistance ability were selected as material. S39 is prone to bolting and very sensitive to high temperature, whereas S24 is bolting resistant and is difficult to bolt even under high temperature. Thus S24 is more attractive and preferred by growers. We conducted morphological, physiological, transcriptomic and proteomic comparisons and characterizations of these two lines and found that GA, auxin, cell cycle related genes and MADS-box genes mediate the bolting and flowering, in which the MADS-box flowering integrators may play the decisive role for regulation of the time of bolting in lettuce.

## Results

### Morphological and Physiological Comparisons of Two Lettuce Lines With Differential Bolting Resistance

**FIGURE 1:**
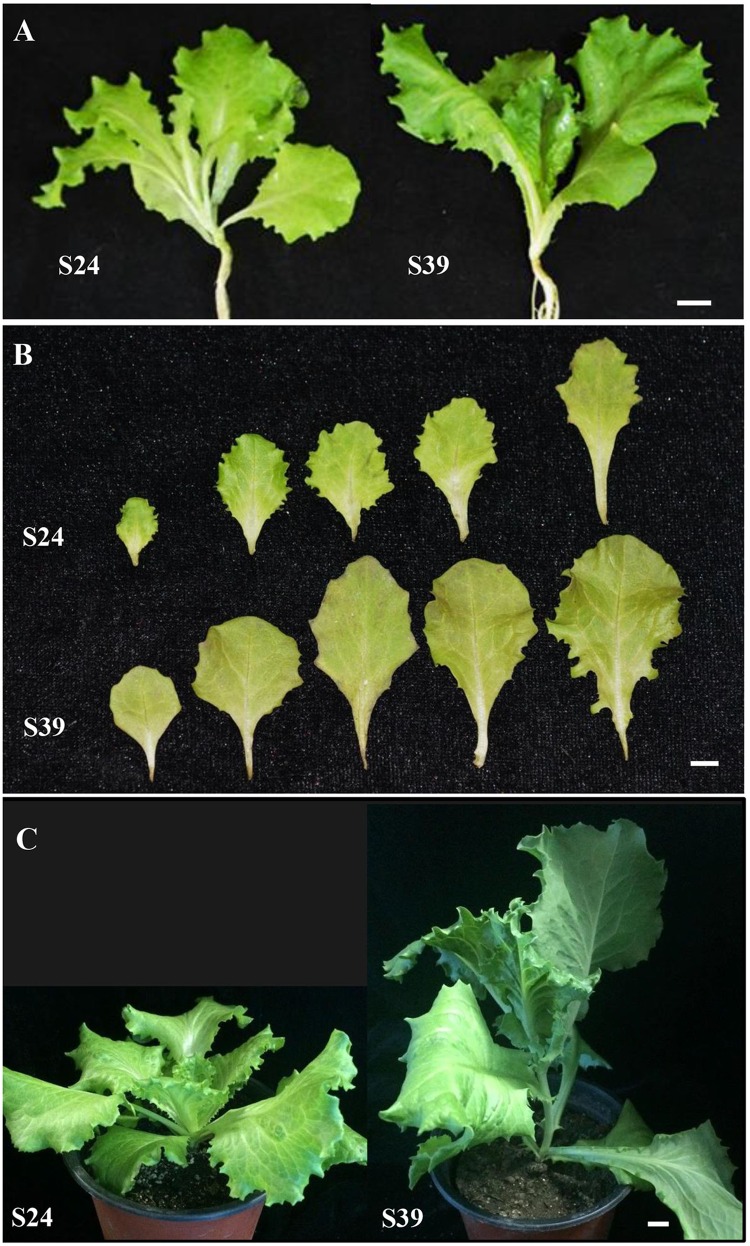
**Morphological characterization of two lettuce lines S24 and S39.** **(A)** Representative images of bolting resistant line S24 and bolting sensitive line S39 at five true leaf stage. **(B)** Leaf size, shape and color differences between S24 and S39 at five true leaf stage. **(C)** Representative images of S24 and S39 at seven true leaf stage grown in the greenhouse. Line S39 was apparently bolting while S24 was not bolting. Bar = 1 cm.

To understand the mechanisms of heat-induced bolting in lettuce, we collected 705 lettuce varieties and planted in the field of the Experimental Station of Beijing University of Agriculture in the summers of 2008 – 2010. Because lettuce is self-pollinated and it is difficult to make crosses, we were unable to generate near-isogenic lines or hybrid populations. Thus, we just chose varieties that were highly similar in appearance but displayed distinct abilities in bolting resistance. Based on this approach, line S24 was chosen as a bolting resistant line and line S39 as a bolting sensitive line. Prior to this study, line S24 and line S39 have been stabilized for six generations through selfing. Both lines S24 and S39 belong to leafy lettuce with serrated leaves (**Figure 1A**). As compared to line S24, line S39 generally grew faster and its leaves were bigger with slight purple (**Figure 1B**). More importantly, S39 is prone to bolting and is very sensitive to high temperature, whereas S24 is bolting-resistant and heat-insensitive. If they are grown in parallel in the summer under high temperature, S39 will bolt 30 days earlier than S24 (**Figure 1C**).

**FIGURE 2:**
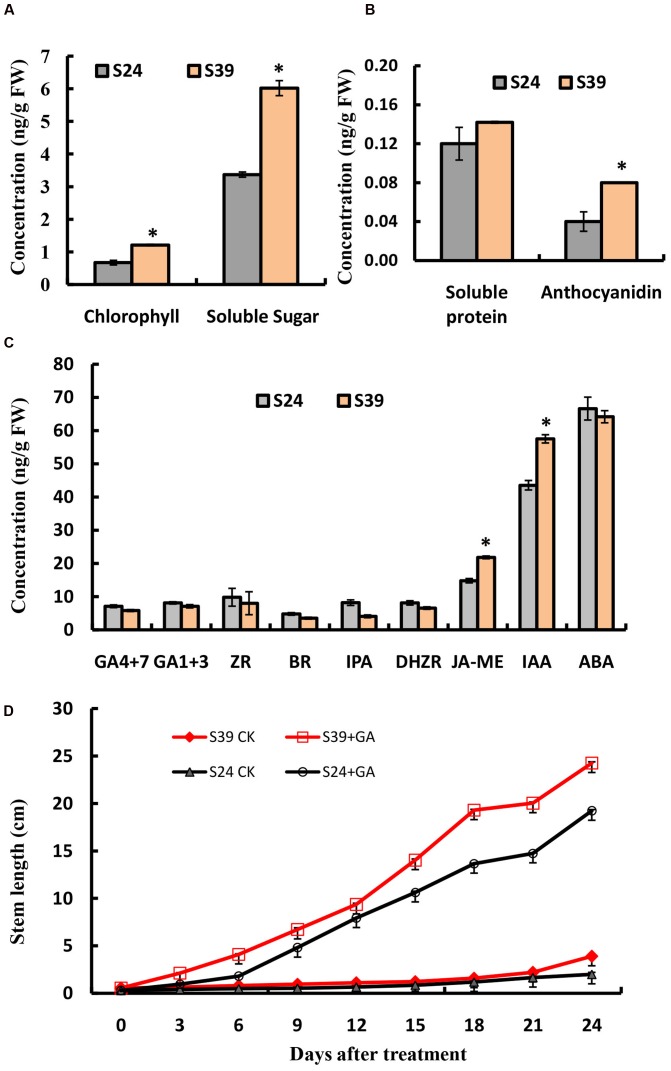
**Physiological measurements of S24 and S39.** **(A,B)** Content of chlorophyll and soluble sugar **(A)**, soluble protein and anthocyanidin **(B)** in the leaves of S24 and S39 at the five true leaf stage. **(C)** Content of endogenous hormones (auxin, cytokinin, gibberellin, jasmonic acid, brassinosteroids, and abscisic acid) in the leaves of S24 and S39 at the five true leaf stage. **(D)** Stem elongation in lines S24 and S39 after exogenous gibberellin for 24 days 3 treatment. **^∗^**Represent significant difference at *P* < 0.05 by student *t*-test.

Next, we compared the content of chlorophyll, soluble sugar, soluble protein and anthocyanidins in the leaves of line S24 and S39. Consistent with its appearance, line S39 has about two fold higher in the content of chlorophyll, soluble sugar and anthocyanidins than line S24, while there is no significant difference in the level of soluble protein between these two lines (**Figures 2A,B**). Phytohormones have been intensively studied for their essential role in plant growth and development (Kepinski, 2006; Durbak et al., 2012). To explore the hormonal difference between line S24 and S39, we measured the levels of gibberellins (GA4 + 7, GA1 + 3), cytokinins (ZR: trans-zeatin riboside; IPA: 3-Indole propionic acid; DHZR: dihydrogen zeatin riboside), brassinosteroid (BR), jasmonic acid (JA-ME), auxin (IAA: 3-Indole acetic acid) and abscisic acid (ABA) in the leaves of line S24 and S39. As shown in **Figure 2C**, the concentrations of auxin (IAA) and jasmonic acid (JA-ME) were significantly higher, and the level of gibberellins (GA4 + 7) and cytokinins were slightly reduced in S39 than S24. All other hormones showed no significant difference between the two lines (**Figure 2C**).

Because endogenous GAs have been previously shown to mediate the heat-induced stem elongation in lettuce (Fukuda et al., 2009), we next explored the effects of exogenous GA treatment on bolting of lines S39 and S24. As shown in **Figure 2D**, GA could dramatically promote bolting in both S39 and S24 lines (**Figure 2D**). In the GA treated plants, S39 and S24 started bolting on days 5 and 7, respectively. In the mock treated plants, S39 started bolting on day 23, while S24 did not bolt during the whole period of experiment (25 days) (**Figure 2D**).

### Transcriptome Analyses of Lines S24 and S39 Through RNA-Seq

To explore genes and gene networks that control the time of bolting in lettuce, we performed RNA-Seq analysis of lines S24 and S39 using the fourth leaf during the five true leaf stage as materials (**Figure 1A**). Three biological replicates were performed for each lettuce line. A total of 166,583,308 paired-end reads were used for *de novo* assembly of the lettuce transcriptome (**Table 1**). After clustering and filtering, we obtained 36,762 lettuce transcripts that were longer than 200 bp. There are 5,901 SwissProt proteins and 8,151 TAIR10 proteins, respectively, that are represented by nearly full-length transcripts, having >80% alignment coverage (**Table 1**). Using fold change ≥2 and false discovery rate (FDR) < 0.05 as cutoff, 5,860 genes were differentially up-regulated and 6,344 genes were differentially down-regulated in S39 versus S24 (**Supplementary Tables S1** and **S2**).

**Table 1:** Summary of transcriptome sequencing data.

Sample	Raw reads (M)	Clean reads (Clean/All)	Mapped reads (Mapped/Clean)	Uniquely mapped reads (Unique/Clean)
S24_rep1	29.34	28.21 (96.15%)	24.86 (88.13%)	20.65 (73.22%)
S24_rep2	28.48	27.41 (96.24%)	24.17 (88.17%)	19.64 (71.64%)
S24_rep3	29.76	28.67 (96.34%)	25.36 (88.45%)	20.82 (72.60%)
S39_rep1	32.15	30.92 (96.17%)	27.35 (88.46%)	22.98 (74.33%)
S39_rep2	28.05	26.99 (96.22%)	23.98 (88.85%)	20.04 (74.25%)
S39_rep3	25.28	24.38 (96.44%)	21.74 (89.17%)	18.17 (74.53%)


### Validation of RNA-Seq Data by Quantitative Real Time RT-PCR (qRT-PCR)

**FIGURE 3:**
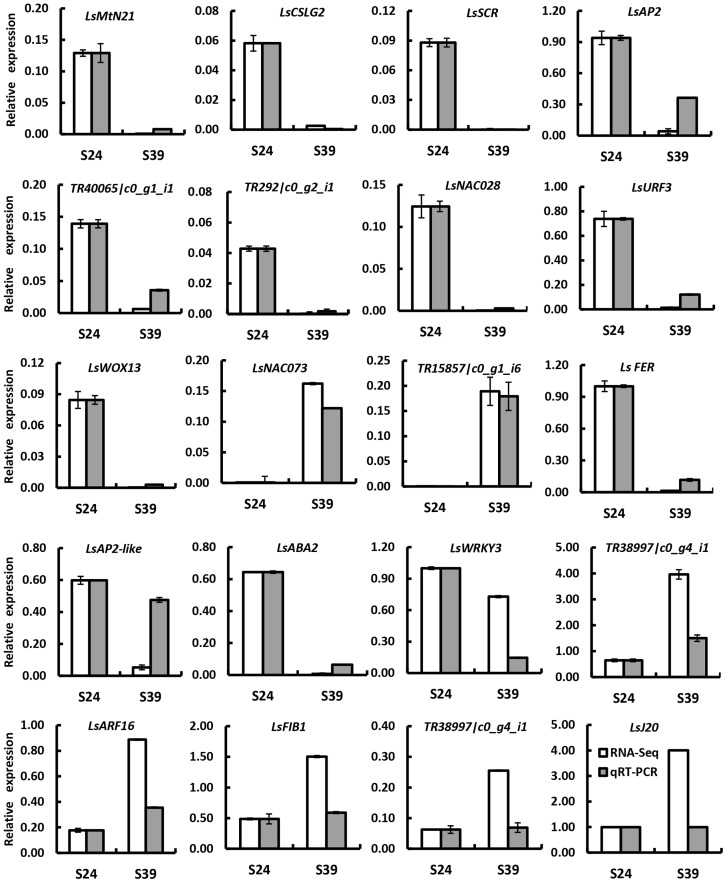
**Verification of differentially expressed genes by qRT-PCR.** Twenty genes were chosen for qRT-PCR validation. The white and gray bars represent the relative expression levels of each gene in the lines S24 and S39 as detected by RNA-Seq and qRT-PCR, respectively. To plot the RNA-Seq data, the gene expression in S24 was set to be the same as that in qRT-PCR, and the relative expression in S39 was calculated using the fold-change as detected by RNA-Seq. The lettuce 18S ribosomal RNA (HM047292.1) was used as an internal standard to normalize the expression data. The bars represent the standard deviation (*n* = 3).

To confirm the differentially expressed genes (DEGs) identified by RNA-Seq, 20 DEGs were randomly chosen for quantitative real time RT-PCR analyses using independently collected samples that were at the same developmental stage as those used for RNA-Seq analysis. Among the 20 selected DEGs, 7 genes showed higher expression and 13 genes displayed lower expression in line S39 according to the RNA-Seq data. As shown in **Figure 3**, 19 out of the 20 genes showed the same expression patterns in the qRT-PCR assays as in the RNA-Seq data. The Pearson’s correlation coefficient between qRT-PCR and RNA-Seq was 0.83, suggesting that the RNA-Seq data were highly reliable.

### Functional Analyses of Differentially Expressed Genes

**FIGURE 4:**
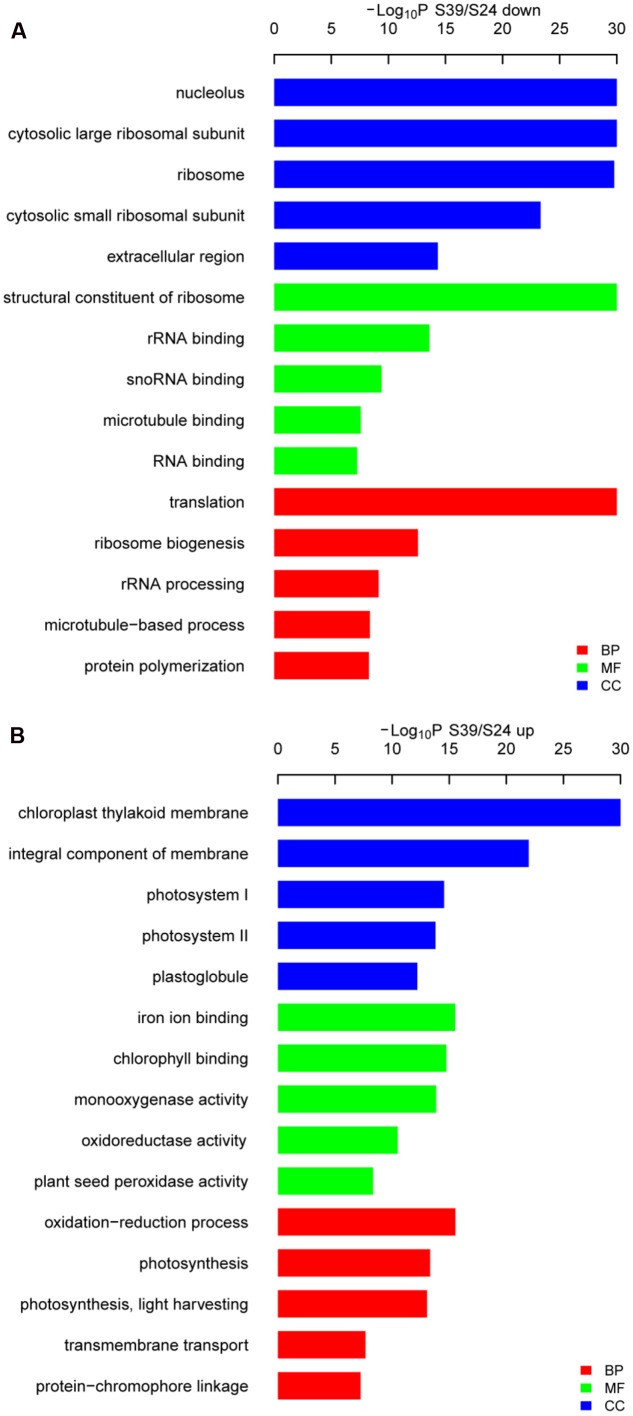
**Gene ontology (GO) analysis of the down-** **(A)** and up-regulated **(B)** genes in line S39 vs. S24. GO terms belong to biological processes (BP), molecular functions (MF), and cellular components (CC) were shown in red, green, and blue, respectively. Only the top five significantly enrich GO terms (*p* < 0.05) were shown for each group. GO terms are sorted based on *p*-values.

To identify the significant changes in biological process (BP) and molecular function between S24 and S39, Gene Ontology (GO) annotation was performed using the Trinotate through BLAST search against the well-annotated protein sequences (SwissProt) and protein domain identification database (PFAM). 22,014 transcripts (59.9% of the assembled transcripts) were assigned with at least one GO term. Next, the up- and down-regulated DEGs between S39 and S24 were utilized for the GO term enrichment analysis. For genes that were down-regulated in line S39, the top four significantly enriched GO terms were “translation,” “ribosome biogenesis,” “rRNA processing” and “microtubule-based process,” respectively, in the BP category (**Figure 4A**), suggesting that protein synthesis is less active in line S39 as compared to line S24. This is consistent with the physiological data that despite the content of soluble sugar was much higher in line S39, the level of soluble protein was about the same in lines S39 and S24. A total of 45 genes implicated in microtubule-based process were significantly down-regulated in line S39 (**Supplementary Table S5**). For example, TR33842| c1_g1_i2, a gene that encodes a putative kinesin motor domain containing protein, was 84.4 fold (LogFC = -6.4) down-regulated in line S39. The expression of TR37794| c0_g1_i1, a putative microtubule associated gene, was 24.3 fold (LogFC = -4.6) lower in line S39 (**Supplementary Table S5**). In the up-regulated DEGs, the top two enriched GO terms were “oxidation-reduction process” and “photosynthesis” in the BP group (**Figure 4B**), indicating the enhanced photosynthesis and oxidation-reduction process in line S39. Given that anthocyanins have been shown to possess strong antioxidant properties in plants (Solomon et al., 2006; Burdulis et al., 2009), the induced gene expression involved in oxidation-reduction process was consistent with the increased anthocyanidins in line S39 (**Figure 2B**). Similarly, the elevated gene expression implicated in photosynthesis matched perfectly with the higher levels of chlorophyll and soluble sugar in line S39 (**Figure 2A**).

**FIGURE 5:**
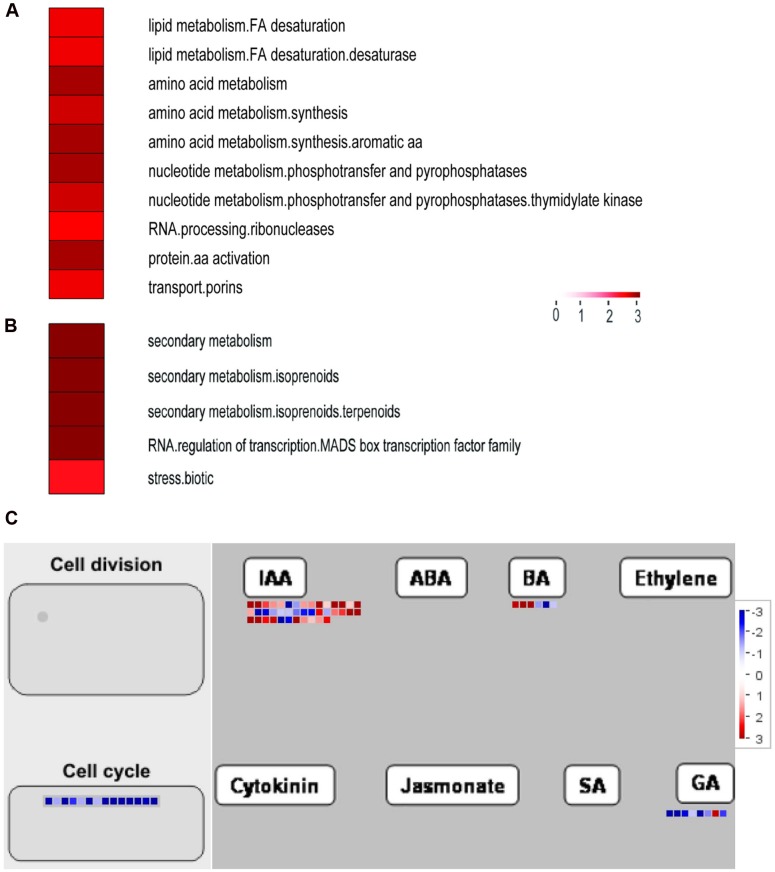
**Functional categorizations of the differentially expressed genes in line S39 vs. S24 using the MapMan software.** **(A,B)** The significantly enriched functional categories in the down- **(A)** and up-regulated **(B)** genes between S39 and S24. Categories are sorted based on adjusted *p*-values. **(C)** Cellular response and hormone regulation overview of the differentially expressed genes between S39 and S24. Each colored dot represents one gene. The color represents the log_2_ fold changes, blue indicates a decrease (lower expression in S39), and red indicates an increase (higher expression in S39).

Next, functional categorizations of the DEGs between lines S24 and S39 were performed using the software MapMan (**Figure 5**). In agreement with results in the GO enrichment analysis, genes involved in lipid and amino acid metabolisms were significantly enriched in the down-regulated DEGs (**Figure 5A**), whereas genes implicated in secondary metabolism, MADS-box transcription factor and biotic stress were greatly enriched in the up-regulated DEGs (**Figure 5B**). A total of 12 MADS-box transcription factors were dramatically induced in line S39, and all of them have been shown to promote flowering in *Arabidopsis* (**Supplementary Table S6**) (Komeda, 2004; Wellmer and Riechmann, 2010). For example, the expression of well-known flowering integrators *LsSOC1* (TR9802| c1_g1_i2) and *LsAP1* (TR33988| c0_g1_i1) were 256 (LogFC = 8) and 548.7 (LogFC = 9.1) fold respectively, higher in line S39 than S24 (**Supplementary Table S6**) (Moon et al., 2003; Lee and Lee, 2010), supporting the bolting sensitive phenotype in line S39 (**Figure 1**). Further, cellular response overview of the DEGs showed that genes related to cell cycle, but not cell division, were significantly repressed in line S39, which is consistent with the down-regulation of microtubule-related genes (**Supplementary Table S6**) and suggests that cell growth is terminated later in line S39, resulting in enlarged leaf size (**Figure 1B**). In addition, hormone regulation overview of the DEGs demonstrated that most auxin-related genes were significantly up-regulated, while gibberellin-related genes were largely down-regulated in line S39 (**Figure 5C**). In particular, 28 out of 41 auxin-related genes and 7 out of 8 GA-related genes were up-regulated and down-regulated, respectively, in line S39 (**Supplementary Table S7**, **Table 2**), which explained the increased auxin level and decreased GA content in line S39 (**Figure 2C**).

**Table 2:** List of GA-related genes that were differentially expressed in lettuce line S39 vs. S24.

Gene ID	Putative function	*Arabidopsis* homolog	log_2_FC	FDR
TR35751| c0_g1_i1	GASR6 - Gibberellin-regulated family protein	AT1G75750.1	2.8	9.4E-20
TR2500| c0_g1_i1	GASR5- Gibberellin-regulated family protein	AT1G74670.1	-6.1	3.0E-52
TR5731| c1_g1_i1	GASR5 - Gibberellin-regulated family protein	AT1G74670.1	-6.9	5.9E-19
TR5731| c1_g2_i1	GASR5 - Gibberellin-regulated family protein	AT1G74670.1	-2.7	1.8E-20
TR9620| c0_g1_i1	GASR10 - Gibberellin-regulated family protein	AT1G74670.1	-1.1	1.5E-06
TR16738| c0_g1_i1	Gibberellin-regulated family protein	AT5G14920.1	-8.5	1.1E-14
TR32173| c0_g2_i1	NA	NA	-1.7	6.3E-07
TR42959| c0_g1_i1	GASR3 - Gibberellin-regulated family protein	AT5G59845.1	-2.2	3.3E-08


### Identification of Differentially Accumulated Proteins from the Leaves of Lines S39 and S24 upon Heat Treatment

**FIGURE 6:**
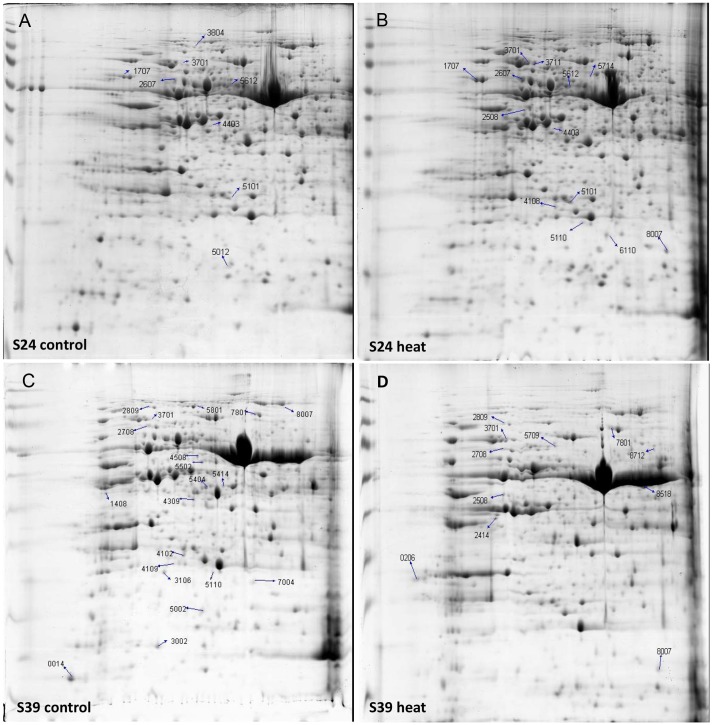
**2-DE gel analysis showed the differentially accumulated proteins from the leaves of line S39 and line S24 upon heat treatment.** **(A)** Line S24 under control treatment (26°C). **(B)** Line S24 under heat treatment (42°C). **(C)** Line S39 under control treatment (26°C). **(D)** Line S39 under heat treatment (42°C). The differentially accumulated protein spots were calculated as heat vs. control treatment, and numbered and indicated by arrows for each lettuce line.

To explore the heat response difference of lines S24 and S39 at the protein level, the two lettuce lines at the five true leaf stage were subjected to heat treatment for 48 h at 42°C, and the corresponding lettuce seedlings kept at 26°C were used as control. 2-DE gel analyses were performed and differentially accumulated proteins were identified by comparing the heat treated samples with the control samples for each lettuce line. Three biological replicates were performed for each treatment. More than 100 protein spots were reproducibly detected on 2-DE gels using PDQuest software. After enzyme solution treatment, each protein point was processed by MALDI-TOF mass spectrometry for peptide mass fingerprint (PMF) analysis. Proteins were screened based on three criteria: (1) the same protein point was detected in all three replications; (2) Protein expression quantities changed at least two folds between the heat treatment sample and the control sample; (3) protein quantity showed statistically significant change (*P* < 0.05). Based on these criteria, a total of 103 protein points were screened and 30 protein points were obtained (**Figure 6**). Six out of the 30 proteins were commonly up-regulated upon heat treatment in both lines S39 and S24, including two putative heat shock cognate protein 70-1 and two TCP-1/cpn60 chaperonin family protein (Prasad and Stewart, 1992; Sung and Guy, 2003) (**Supplementary Table S3**). Nine and five proteins were specifically induced in S24 and S39, respectively, in which proteins involved in protein synthesis were uniquely up-regulated in line S24, while metabolism related proteins were uniquely up-regulated in line S39. For example, four genes involved in translation elongation were specifically up-regulated in line S24 (**Supplementary Table S3**). Ten out of the 30 proteins were repressed by heat treatment (**Supplementary Table S3**). Interestingly, genes that encode19 out of the 30 differentially accumulated proteins showed differential expression in the RNA-Seq data (**Supplementary Table S3**), suggesting our proteomic data were consistent with our RNA-Seq data.

## Discussion

### Photosynthesis, Protein Synthesis and Stress Response Represent the Intrinsic Differences between Lines S39 and S24

Photosynthesis is a fundamental process that converts light energy into chemical energy, and supplies all organic compounds and most of the energy essential for life on earth (Bryant and Frigaard, 2006). Photosynthesis is very sensitive to heat stress (Berry and Bjorkman, 1980), and there are at least three major stress-sensitive sites in the photosynthetic machinery including photosystem II with its electron donor and acceptor (Pospisil and Tyystjarvi, 1999; Wen et al., 2005), carbon fixation with the key enzymes Rubisco and Rubisco activase (Crafts-Brandner and Salvucci, 2000; Salvucci and Crafts-Brandner, 2004), and chlorophyll thylakoid membrane (Morgan-Kiss et al., 2002). Chlorophyll is the major photosynthetic pigment that captures sunlight for photosynthesis. Anthocyanins, on the other hand, mainly possess two functions in plants: one is to protect cells from high-light damage by absorbing blue-green and ultraviolet light, and the other is to act as antioxidants by scavenging free radicals (Gould et al., 2002; Chen et al., 2013). The jasmonic acid (JA) is well known to mediate biotic and abiotic stress responses in plant (Creelman and Mullet, 1995). In this study, we found that the content of chlorophyll, soluble sugar, anthocyanidin and JA was higher in the heat-sensitive line S39 (**Figures 2A,B**). Consistently, genes related to oxidation-reduction, photosynthesis and biotic stress were significantly up-regulated in line S39 (**Figures 4B** and **5B**). On the other hand, GO terms “translation” and “ribosome biogenesis” were highly enriched and related genes were up-regulated in the heat insensitive line S24 (**Figure 4A**). Consistently, the relative soluble protein (soluble protein/soluble sugar) was higher in S24 (**Figures 2A,B**). In addition, proteomic analysis of lines S24 and S39 upon heat treatment showed that proteins involved in protein synthesis were uniquely up-regulated in line S24, while metabolism related proteins were uniquely up-regulated in line S39 (**Figure 6**). Considering that lines S24 and S39 were from different genetic backgrounds, difference in photosynthesis, protein synthesis and stress response may be resulted from their genetic differences.

### Gibberellins and Auxin Mediate Stem Elongation and Organ Size in Lettuce

Phytohormones such as gibberellins and auxin have been documented to regulate flowering transition and organ size (Brian, 1958; Davies, 2003). GA promote flowering of Arabidopsis by activating the LEAFY promoter, and crosstalk between photoperiod and vernalization pathways (Zanewich and Rood, 1995; Blazquez et al., 1998; Yu et al., 2004). In lettuce, expression of a putative GA 3-oxidase gene, LsGA3ox1, is significantly up-regulated by high temperature (Fukuda et al., 2009). GAs have also been shown to promote cell elongation through repressing DELLA proteins, and thus gibberellic acid-deficient *Arabidopsis* mutant plants exhibited reduced petal growth (Cheng et al., 2004). Consistently, here we found that exogenous GA treatment can dramatically promote bolting in both S39 and S24 lines. S39 and S24 started bolting on days 5 and 7, respectively, upon GA treatment (**Figure 1**), but the content of endogenous GA was only slightly reduced in S39 (**Figure 2**), suggesting that the huge difference in bolting time of lines S39 and S24 may not be caused by the action of GA. In addition, the leaf size was bigger and the endogenous auxin was higher in S39 (**Figures 1** and **2**). Accordingly, genes related to the auxin pathway were mostly up-regulated in S39 (**Figure 5**). Given that auxin was shown to regulate cell proliferation and cell expansion, and mutation of genes implicated in auxin actions such as *AUXIN RESPONSE FACTOR8* and *PETAL LOSS* resulted in changes of petal size (Griffith et al., 1999; Varaud et al., 2011), it is plausible to speculate that the enlarged leaf size was due to the elevated auxin activity in S39.

### Flowering Integrators May Play a Decisive Role in the Bolting Time Regulation in Lettuce

Flowering integrators such as *FT* and *SOC1* have been well documented to regulate the flowering time in many species (Mouradov et al., 2002; Parcy, 2005). SOC1 interacts with multiple MADS box proteins, including FUL, AP1 and AGL24, and promotes flowering in *Arabidopsis* (Komeda, 2004; Liu et al., 2008; Melzer et al., 2008; Torti and Fornara, 2012; Balanza et al., 2014). Overexpression of *DoSOC1*, an ortholog of *Arabidopsis SOC1*, promotes flowering in the orchid *Dendrobium* (Ding et al., 2013). However, in the perennial short-day plant woodland strawberry, overexpression of *FvSOC1* inhibits flower initiation under inductive short days, whereas silencing of *FvSOC1* leads to continuous flowering in both short days and long days (Mouhu et al., 2013). In this study, lettuce line S39 is prone to bolting and very sensitive to high temperature. Under normal temperature, S39 generally starts bolting at the seven true leaf stage while S24 will not bolt during the whole growth period (**Figure 1**). When grown in the summer with high temperature, S39 would bolt 30 days earlier than S24 (**Figure 1C**). Consistently, a total of 12 MADS box transcription factors were dramatically induced in line S39 such as putative *LsSOC1, LsAP1, LsFUL* and *LsAGL24* (**Supplementary Table S6**). Particularly, putative *LsSOC1* (TR9802| c1_g1_i2) were 256 fold (LogFC = 8) up-regulated in line S39 (**Supplementary Table S6**), suggesting that the flowering integrator *LsSOC1* may play a decisive role in the differential bolting resistance between S39 and S24. Future functional studies using genetic transformation in lettuce and expression analyses of *LsSOC1* in natural populations would be promising to dissect the precise regulation of bolting time in lettuce.

## Materials and Methods

### Plant Materials

The leafy lettuce (*Lactuca sativa* L.) lines S24 (bolting resistant) and S39 (bolting sensitive) were grown in the Beijing University of agriculture Experimental Station of Beijing under standard greenhouse conditions. Pest control and water management were performed according to standard practices. When the lettuce plants developed the fifth true leaf, the fourth leaves from S24 or S39 lines were collected at the same time on the same day. Leaf samples from 5 different seedlings were pooled together as one biological sample. Three biological replicates from each line were used for RNA-Seq analyses. Samples were immediately frozen in liquid nitrogen and stored at -80°C until further use.

### Determination of Chlorophyll Levels

About 0.5 g leaf tissue (the fourth leaf in the fifth-leaf stage) was smashed and transferred into a 50 ml tube. Chlorophyll was extracted with 25 ml 95% (v/v) ethanol, and the resultant supernatant was measured using a spectrophotometer (UV-2102C, YOUNIKE, China) at 649 nm (A_649_), 665 nm (A665), and 470 nm (A_470_) wavelengths, respectively. Contents of chlorophyll were calculated as described Zaharieva and Goltsev (2003).

### Measurement of the Anthocyandinin

About 0.2 g fresh leaf (the fourth leaf in the fifth-leaf stage) was collected and placed in a 50 ml beaker, and then 10 ml 2% hydrochloric acid methanol solution was added to soak at the room temperature with no light. After 2 h filtration with 2% hydrochloric acid methanol solution to a 50 ml volumetric flask, and the resultant supernatant was measured using a spectrophotometer (UV-2102C, YOUNIKE, China) at the 530 nm (A530) wavelength.

### Determination of Soluble Protein and Sugar Content

For determination of the soluble protein content, about 1 g fresh leaf tissue (the fourth leaf in the fifth-leaf stage) was collected and extracted with 5 ml 0.05 mol/L phosphate buffer (pH = 7.8), followed by centrifugation at 4000 *g* for 10 min in 4°C. The resultant supernatant was admixed with 5 ml coomassie brilliant blue G-250-protein reagent, and the optical density (OD) value at the 595 nm wavelength was measured using a spectrophotometer (UV-2102C, YOUNIKE, China) (Jiang and Huang, 2002). For determination of the soluble sugar content, about 1 g fresh sample was grounded and boiled in water bath. Soluble sugars were measured using the traditional anthrone colorimetric method by measuring the OD value at the 630 nm wavelength as described Hu et al. (2009). Student *t*-test was used to explore whether S24 and S39 showed significant difference in physiological measurements (chlorophyll, soluble sugar, soluble protein, anthocyanin) using *p* < 0.05 as significance cutoff.

### Endogenous Hormone Measurement

To determine the contents of auxin, cytokinin, GA, JA, BR, and ABA in S24 and S39, leaf tissues (the fourth leaf in the fifth-leaf stage) were collected and hormone measurements were performed using enzyme-linked immunosorbent assays (ELISA) as previously described (Maldiney et al., 1986; Abdala et al., 1996; Cui et al., 2005; Swaczynová et al., 2007). Standard auxin (IAA), gibberellins (GA1 + 3, GA4 + 7), I3-Indole propionic acid, *trans*-zeatin riboside, dihydrogen zeatin riboside, ABA, JA, and BR (Sangon Biotech Co. Ltd, Shanghai, China) were used for calibration. Three biological replicates and three technical replicates were performed for each hormone measurement, and the results were presented as mean ± SE of three biological replicates. Student *t*-test was used to test whether S24 and S39 showed significant difference in concentration of each endogenous hormone using *p* < 0.05 as significance cutoff.

### Exogenous Gibberellin Treatment

During the trial experiment, gibberellin 3 with different concentrations (25 mg/L, 50 mg/L, 100 mg/L, and 200 mg/L) was sprayed to S24 and S39 seedlings, and 50 mg/L gibberellin 3 was chosen for formal exogenous gibberellin treatment. Plants at the fifth true leaf stage with uniform growth were selected from S24 and S39 lines and sprayed with 50 mg/L gibberellin. Water was used as the negative control. Twelve plants were used for each treatment, and the stem length was measured every day since treatment for 3 weeks.

### RNA Extraction and Quality Test

The fourth leaf in the fifth-leaf stage was used for RNA extraction. Total RNA was extracted using the RNA extraction kit (Aidlab, China). RNA concentration was measured by Qubit RNA Assay Kit in Qubit 2.0 Flurometer (Life Technologies, Camarillo, CA, USA), and RNA integrity was evaluated by RNA Nano 6000 Assay Kit of the Bioanalyzer 2100 system (Agilent Technologies, Santa Clara, CA, USA). Only RNA samples that passed the quality tests were chosen for RNA-Seq analyses.

### RNA-Seq Library Construction and Sequencing

RNA-Seq library construction was performed following the manufacturer’s instructions of the NEBNext Ultra Directional RNA Library Prep Kit for Illumina (NEB, Ispawich, MA, USA) and four index codes were added to attribute sequences to different samples (Wang et al., 2009). RNA-seq libraries were sequenced on an Illumina HiSeq 2000 platform to generate 100 bp pair-ended reads. Sequencing data were deposited to the Sequence Read Archive (SRA) at the National Center for Biotechnology Information (NCBI) with accession number SRP076512.

### Bioinformatics Analysis of RNA-Seq Data

Raw sequencing reads were quality checked for low quality regions and adapter sequences using SolexaQA_v.2.2 and cutadapt tool (v1.4.2) (Martin, 2011). Regions with quality score below 17 were trimmed using DynamicTrim and reads with a remaining length less than 25 bp were discarded. The Trinity software package (v2.0.6) was used for *de novo* transcriptome assembly from the RNA-seq data (Grabherr et al., 2011). The resulting pre-assembled transcripts were refined according to the methods described by Ranjan et al. (2014). Transcripts were filtered out with an abundance cutoff value of 1 and the remained transcripts were clustered using CD-HIT-EST with parameters -c 0.95 -n 8. The quality of the assembled transcriptome was evaluated by examining the number of assembled transcripts that appear to be full-length or nearly full-length by comparing to the proteins in SwissProt and TAIR10. RNA-Seq by expectation maximization (RSEM), which allows for an assessment of transcript abundances based on the mapping of RNA-Seq reads to the assembled transcriptome, was used for transcript abundance estimation of the *de novo*-assembled transcripts (Li and Dewey, 2011). Transcript abundance values were fed to EdgeR (Robinson et al., 2010) to identify genes that were differentially expressed in two lines. The genes with at least two fold change in expression and a false discovery rate (FDR) < 0.05 were considered to be differentially expressed.

### Gene Ontology Term Enrichment Analysis

The assembled transcripts were annotated by Trinotate with GO terms describing the biological processes, molecular functions, and cellular components (Gotz et al., 2008). The GO annotation report was filtered using a cut-off e-value of 1E-5. After annotation, the genes with an expression level of at least 1 count per million (CPM) in at least three samples were retained for further analysis. The R package edgeR was used to identify the differential expressed genes (DEGs) using the threshold of at least two-fold change in expression and the FDR of less than 0.05 (Robinson et al., 2010). The up- and down-regulated DEGs were then conducted for GO enrichment analysis respectively, using TopGO (Alexa et al., 2006). Adrian Alexi’s improved weighted scoring algorithm and Fisher’s exact test were used to determine the significance of GO term enrichment. Significantly enriched GO terms were identified as those with a *p*-value less than 0.05.

### MapMan Analysis of DEGs

The online web tool Mercator (Lohse et al., 2014) was used to obtain the lettuce protein annotation mapping file for MapMan with a blast cutoff of 50 and then used the Java software MapMan to assign functional categorizations to the differentially expressed genes (Thimm et al., 2004). Fisher’s exact test was used to examine whether a functional category was significantly overrepresented in our selected genes against the set of all genes with MapMan annotations, the Benjamini Hochberg method was used to adjust *p*-values for multiple testing.

### Quantitative Real-Time RT-PCR

Quantitative real-time RT-PCR analyses were performed with independently generated samples from S24 and S39 lines at the same developmental stage. cDNAs were reverse-transcribed from 3 μg total RNA using the PrimeScript RT reagent Kit (Takara, Da Lian, China), and qRT-PCR was performed with an ABI PRISM 7500 Real-Time PCR System (Applied Biosystems, USA). The lettuce 18S ribosomal RNA (GeneBank number: HM047292.1) was used as an internal reference to normalize the expression data. Each qRT-PCR experiment was performed with three biological replicates and technical replicates (3 × 3). The relative expression of each gene was calculated using the 2^-ΔΔCt^ method (Livak and Schmittgen, 2001) and standard deviation was calculated among three biological replicates. The primer sequences are listed in **Supplemental Table S4**.

### Heat Treatment and 2-DE Gel Electrophoresis

When the lettuce plants developed the fifth true leaf, S24 and S39 seedlings were moved to the growth chamber for heat treatment at 42°C for 48 h. After treatment, the fourth leaves were cut off and frozen in liquid nitrogen, and stored at -80°C for protein and enzyme extraction. Seedlings moved to the growth chamber at 26°C for 48 h were used as a control. All experiments were performed with three biological replicates.

Protein extraction and two-dimensional electrophoresis were carried out according to Shen et al. (2003). The second dimension SDS-PAGE was performed with 15% resolving gels and 5% stacking gels (130 mm × 140 mm × 1 mm in total). The gels were stained with 0.1% Coomassie brilliant blue (CBB) R-250.

## Additional Information

Supplementary information accompanies this paper at http://www.nature.com/Scientificreports.

## Author Contributions

YH, ZC, SF, and XZ conceived and designed the experiments. YH, ZC, XJ, KN, and XL performed the experiments. SL, RL, and XZ analyzed the data. YH, ZC, RL, QW, and XZ wrote the paper. All authors read and approved the final manuscript.

## Conflict of Interest Statement

The authors declare that the research was conducted in the absence of any commercial or financial relationships that could be construed as a potential conflict of interest.

## Footnotes

1 http://faostat3.fao.org/browse/Q/QC/E
