# Supplementary material for: MADS-Box Genes and Gibberellins Regulate Bolting in Lettuce (Lactuca sativa L.)
Source: Front Plant Sci. 2016 Dec 16;7:1889. doi: 10.3389/fpls.2016.01889 (PMC5159435; doi:10.3389/fpls.2016.01889)
Supplement: Supplementary file 5 [file Table_5.DOC]

**Supplemental Table S5. List of selected** **microtubule-related genes that were differentially down-regulated in lettuce line S39 vs S24**

| **Gene ID** | **Putative function** | **Arabidopsis homologue** | **log_2_FC** | **FDR** |
| --- | --- | --- | --- | --- |
| TR33842|c1_g1_i2 | Kinesin motor domain containing protein | AT5G06670.1 | -6.4 | 3.1E-36 |
| TR41506|c0_g1_i1 | Kinesin motor protein-related | AT1G63640.1 | -3.1 | 4.3E-30 |
| TR43050|c0_g1_i1 | Expressed protein | AT1G74160.1 | -2.6 | 1.9E-26 |
| TR21044|c1_g1_i1 | Microtubule associated protein | AT4G26760.1 | -2.3 | 1.0E-25 |
| TR39455|c0_g1_i1 | Kinesin motor domain containing protein | AT2G47500.1 | -2.4 | 2.8E-24 |
| TR42930|c0_g1_i1 | Kinesin motor domain containing protein | AT3G45850.1 | -2.5 | 3.0E-23 |
| TR21978|c2_g1_i2 | Kinesin motor domain containing protein | AT3G16060.1 | -2.3 | 9.6E-22 |
| TR30319|c0_g1_i1 | Expressed protein | AT5G15580.1 | -3.8 | 3.1E-21 |
| TR41321|c0_g1_i1 | Kinesin motor domain containing protein | AT4G14330.1 | -4.9 | 1.0E-20 |
| TR15879|c1_g2_i1 | ATMAP70 protein | AT1G68060.1 | -3.3 | 2.9E-19 |
| TR3558|c0_g2_i1 | ATEB1A-like microtubule associated protein | AT5G67270.1 | -4.0 | 5.3E-18 |
| TR8179|c0_g1_i2 | Kinesin motor domain containing protein | AT5G27550.1 | -4.0 | 5.3E-17 |
| TR37417|c0_g1_i1 | Kinesin motor domain containing protein | AT3G20150.1 | -3.7 | 7.0E-16 |
| TR15879|c2_g1_i1 | ATMAP70 protein | AT1G68060.1 | -1.5 | 2.7E-15 |
| TR22505|c0_g2_i1 | Kinesin motor domain containing protein | AT5G60930.1 | -5.6 | 5.7E-15 |
| TR26221|c0_g2_i1 | Kinesin motor domain containing protein | AT2G28620.1 | -4.9 | 5.8E-15 |
| TR12207|c0_g1_i1 | Microtubule associated protein | AT5G55230.2 | -3.2 | 9.4E-15 |
| TR6935|c0_g1_i1 | Kinesin motor domain containing protein | AT1G18550.1 | -2.9 | 4.1E-13 |
| TR31587|c1_g1_i1 | HEAT repeat family protein | AT1G27210.1 | -1.7 | 1.5E-12 |
| TR26306|c0_g2_i1 | Kinesin motor domain containing protein | AT2G36200.2 | -3.9 | 1.5E-12 |
| TR33842|c1_g1_i1 | Kinesin motor domain containing protein | AT5G06670.1 | -1.6 | 1.8E-12 |
| TR6625|c0_g2_i1 | HEAT repeat family protein | AT1G27210.1 | -1.5 | 2.2E-12 |
| TR10801|c1_g1_i1 | Expressed protein | AT1G74160.1 | -2.0 | 5.7E-12 |
| TR5273|c0_g1_i1 | Microtubule associated protein | AT5G51600.1 | -3.1 | 5.7E-12 |
| TR16044|c0_g1_i1 | Microtubule associated protein | AT4G26760.1 | -1.4 | 2.0E-11 |
| TR15879|c0_g1_i1 | ATMAP70 protein | AT2G01750.2 | -3.0 | 2.1E-11 |
| TR37794|c0_g1_i1 | Microtubule associated protein | AT5G51600.1 | -4.6 | 2.2E-11 |
| TR26306|c0_g2_i2 | Kinesin motor domain containing protein | AT2G36200.2 | -4.3 | 2.4E-11 |
| TR21845|c0_g2_i1 | HEAT protein | AT2G35630.1 | -1.3 | 5.7E-11 |
| TR36626|c0_g5_i3 | HEAT repeat family protein | AT4G27060.1 | -1.7 | 7.9E-11 |
| TR29744|c0_g2_i1 | Kinesin motor domain containing protein | AT1G72250.2 | -2.0 | 1.2E-09 |
| TR42527|c6_g1_i2 | Kinesin motor domain containing protein | AT3G44050.1 | -1.9 | 6.9E-09 |
| TR26476|c0_g1_i1 | Kinesin motor domain containing protein | AT4G27180.1 | -1.1 | 7.2E-09 |
| TR21978|c2_g1_i1 | Kinesin motor domain containing protein | AT3G16060.1 | -1.2 | 9.1E-09 |
| TR37791|c0_g1_i2 | Kinesin motor domain containing protein | AT3G54870.1 | -1.3 | 1.2E-08 |
| TR17799|c0_g1_i1 | Microtubule associated protein | AT1G27920.1 | -2.3 | 2.7E-08 |
| TR2353|c0_g1_i1 | FYVE zinc finger domain containing protein | AT5G12350.1 | -1.8 | 5.8E-08 |
| TR12976|c0_g2_i1 | Kinesin motor domain containing protein | AT1G01950.3 | -1.1 | 1.0E-07 |
| TR17136|c0_g1_i1 | HEAT repeat family protein | AT4G27060.1 | -1.8 | 1.1E-07 |
| TR1853|c0_g1_i1 | Kinesin motor domain containing protein | AT2G22610.1 | -2.7 | 1.1E-07 |
| TR15879|c6_g1_i1 | ATMAP70 protein | AT1G68060.1 | -1.5 | 2.5E-07 |
| TR26221|c0_g1_i1 | Kinesin motor domain containing protein | AT2G28620.1 | -2.1 | 1.0E-06 |
| TR29108|c0_g1_i1 | HEAT repeat family protein | AT4G27060.1 | -1.4 | 4.2E-06 |
| TR36682|c0_g1_i1 | Kinesin motor domain containing protein | AT3G23670.1 | -1.3 | 7.8E-05 |
| TR15879|c8_g1_i1 | ATMAP70 protein | AT1G24764.1 | -1.0 | 1.7E-02 |

**Supplemental Table S6. List of MADS-box genes that were differentially up-regulated in lettuce line S39 vs S24**

| **Gene ID** | **Putative function** | **Arabidopsis homologue** | **log_2_FC** | **FDR** |
| --- | --- | --- | --- | --- |
| TR9802|c1_g1_i2 | SOC1, AGAMOUS-like 20 | AT2G45660 | 8.0 | 8.6E-67 |
| TR9802|c1_g1_i3 | SOC1, AGAMOUS-like 20 | AT2G45660 | 7.7 | 4.4E-49 |
| TR9802|c1_g1_i5 | SOC1, AGAMOUS-like 20 | AT2G45660 | 7.2 | 3.3E-82 |
| TR10008|c1_g1_i1 | AGL24, K-box region and MADS-box TF | AT4G24540 | 1.2 | 1.1E-03 |
| TR30354|c0_g1_i1 | SOC1, AGAMOUS-like 20 | AT2G45660 | 5.7 | 3.1E-21 |
| TR33988|c0_g1_i1 | AP1, K-box region and MADS-box TF | AT1G69120 | 9.1 | 1.1E-17 |
| TR34490|c0_g2_i1 | SEP2, K-box region and MADS-box TF | AT3G02310 | 7.2 | 8.6E-26 |
| TR34986|c0_g1_i1 | FUL, AGAMOUS-like 8 | AT5G60910 | 1.4 | 2.8E-05 |
| TR39708|c0_g1_i1 | AP1, K-box region and MADS-box TF | AT1G69120 | 6.5 | 8.9E-18 |
| TR39708|c0_g1_i2 | AP1, K-box region and MADS-box TF | AT1G69120 | 6.4 | 2.5E-23 |
| TR40725|c2_g2_i1 | FUL, AGAMOUS-like 8 | AT5G60910 | 3.3 | 1.0E-06 |
| TR43255|c0_g1_i1 | FUL, AGAMOUS-like 8 | AT5G60910 | 7.8 | 8.5E-110 |

**Supplemental Table S7. List of auxin-related** genes that were differentially expressed in the lettuce line S39 vs S24

| **Gene ID** | **Putative function** | **Arabidopsis homologue** | **log_2_FC** | **FDR** |
| --- | --- | --- | --- | --- |
| TR615|c0_g1_i1 | Auxin-responsive SAUR gene family member | NA | 3.0 | 1.5E-07 |
| TR615|c0_g2_i1 | Auxin-responsive SAUR gene family member | NA | 3.7 | 5.2E-17 |
| TR1659|c0_g1_i1 | SAUR-like auxin-responsive protein family | AT3G60690.1 | 2.1 | 7.5E-11 |
| TR3130|c0_g1_i1 | Phytochrome-associated protein 2 | AT4G29080.1 | 1.6 | 8.3E-13 |
| TR4292|c0_g1_i1 | SAUR-like auxin-responsive protein family | AT2G24400.1 | 1.4 | 4.1E-04 |
| TR8594|c0_g1_i1 | SAUR-like auxin-responsive protein family | AT3G12830.1 | 1.5 | 1.0E-04 |
| TR8989|c0_g1_i1 | Indole-3-acetic acid inducible 14 | AT4G14550.1 | 1.7 | 4.2E-09 |
| TR9923|c0_g1_i1 | SAUR-like auxin-responsive protein family | AT3G60690.1 | 4.0 | 3.0E-49 |
| TR10131|c0_g1_i1 | NA | NA | 1.0 | 3.7E-03 |
| TR10762|c0_g3_i1 | SAUR-like auxin-responsive protein family | AT4G38840.1 | 4.4 | 2.8E-32 |
| TR10762|c0_g3_i5 | SAUR-like auxin-responsive protein family | AT4G38840.1 | 9.1 | 3.7E-145 |
| TR13664|c0_g2_i1 | AUX/IAA transcriptional regulator family protein | AT1G04550.2 | 1.1 | 3.1E-04 |
| TR13776|c2_g1_i1 | SAUR-like auxin-responsive protein family | AT4G00880.1 | 4.5 | 9.9E-46 |
| TR16572|c0_g1_i1 | Auxin response factor 2 | AT5G62000.1 | 1.4 | 1.6E-12 |
| TR26467|c0_g1_i1 | SAUR-like auxin-responsive protein family | AT1G75590.1 | 2.5 | 7.2E-17 |
| TR30373|c0_g1_i2 | SAUR-like auxin-responsive protein family | AT4G38840.1 | 1.8 | 7.6E-08 |
| TR31735|c2_g1_i1 | SAUR-like auxin-responsive protein family | AT5G50760.1 | 2.2 | 1.7E-06 |
| TR32505|c0_g1_i4 | Auxin-responsive GH3 family protein | AT4G03400.1 | 3.5 | 8.9E-25 |
| TR32505|c0_g1_i5 | Auxin-responsive GH3 family protein | AT4G03400.1 | 3.4 | 1.5E-18 |
| TR33848|c4_g2_i1 | SAUR-like auxin-responsive protein family | AT4G38840.1 | 3.2 | 1.7E-37 |
| TR33848|c4_g2_i2 | SAUR-like auxin-responsive protein family | AT4G38840.1 | 3.9 | 2.3E-20 |
| TR33848|c4_g3_i1 | SAUR-like auxin-responsive protein family | AT4G38840.1 | 2.4 | 5.6E-19 |
| TR34172|c0_g1_i1 | NA | NA | 2.8 | 2.6E-07 |
| TR37757|c0_g1_i1 | SAUR-like auxin-responsive protein family | AT4G38840.1 | 3.0 | 2.2E-13 |
| TR37947|c0_g1_i3 | AUX/IAA transcriptional regulator family protein | AT1G04250.1 | 1.7 | 7.0E-05 |
| TR38226|c0_g1_i1 | Indoleacetic acid-induced protein 16 | AT3G04730.1 | 1.1 | 1.8E-04 |
| TR38238|c0_g1_i1 | Auxin-responsive GH3 family protein | AT2G46370.4 | 1.6 | 2.0E-04 |
| TR43109|c1_g1_i1 | SAUR-like auxin-responsive protein family | AT4G38840.1 | 2.5 | 9.0E-18 |
| TR4729|c0_g1_i1 | SAUR-like auxin-responsive protein family | AT5G18050.1 | -6.7 | 2.9E-13 |
| TR7842|c0_g1_i1 | indole-3-acetic acid inducible 14 | AT4G14550.1 | -1.5 | 6.6E-13 |
| TR17259|c0_g2_i1 | SAUR-like auxin-responsive protein family | AT1G75590.1 | -2.9 | 1.1E-14 |
| TR21207|c0_g1_i1 | Transcriptional factor B3 family protein | AT1G19850.1 | -2.7 | 7.5E-10 |
| TR21952|c1_g2_i1 | Auxin response factor 9 | AT4G23980.1 | -1.5 | 2.1E-04 |
| TR21952|c3_g2_i3 | Auxin response factor 9 | AT4G23980.1 | -1.1 | 4.5E-07 |
| TR22872|c0_g1_i1 | Phytochrome-associated protein 2 | AT4G29080.1 | -1.2 | 5.0E-08 |
| TR23435|c0_g1_i2 | Syphytochrome-associated protein 2 | AT4G29080.1 | -1.8 | 1.7E-05 |
| TR24286|c0_g1_i1 | Auxin-induced protein 13 | AT2G33310.2 | -2.3 | 1.6E-06 |
| TR26281|c0_g1_i1 | SAUR-like auxin-responsive protein family | AT4G34760.1 | -2.4 | 1.2E-23 |
| TR28752|c0_g1_i1 | NA | NA | -1.4 | 3.5E-05 |
| TR35845|c1_g2_i2 | Auxin response factor 19 | AT1G19220.1 | -3.6 | 1.4E-16 |
| TR37044|c0_g1_i1 | Phytochrome-associated protein 2 | AT4G29080.1 | -2.6 | 3.2E-30 |
